# Using AI Algorithms and Machine Learning in the Analysis of a Bio-Purification Method (Therapeutic Emesis, Known as “Vamana Karma”): Protocol for a Mixed Methods Study

**DOI:** 10.2196/79875

**Published:** 2026-02-03

**Authors:** Pooja Rani, Sumit Kalra, Sachin Singh, Richard David, Ashutosh Ravi Gupta, Anandaraman P V

**Affiliations:** 1 Department of Panchakarma, All India Institute of Ayurveda, Mathura Rd, Gautampuri Awas, Sarita Vihar, New Delhi, Delhi, 110076, India, 91 8851304239; 2 Department of Computer Science and Engineering, Indian Institute of Technology Jodhpur, Jodhpur, Rajasthan, India

**Keywords:** validation, therapeutic emesis, TE, vamana karma, traditional medicine, protocol, artificial intelligence, AI

## Abstract

**Background:**

Therapeutic emesis (TE), known as *vamana karma*, is a classical method of detoxification performed to eliminate vitiated *kapha* (bio-humor governing fluid regulation and structural cohesion of the body in normalcy) ailments from the body. The assessment of this complete process depends on physicians’ visual assessments of vomitus features and patient responses, introducing subjectivity and interobserver variability. Moreover, this method requires more than continuous monitoring; thus, it can sometimes lead to human error, resulting in missed expelled content or complications. We propose an artificial intelligence (AI) model to monitor TE to observe visual changes (ie, patient vomitus content and gestures) to provide better clinical outcomes. This approach is being explored for the first time in the traditional system of medicine.

**Objective:**

This study aims to develop and validate an AI-assisted digital framework for the objective evaluation of TE via (1) automatic vomitus detection, (2) content classification, (3) number of bouts expelled, (4) facial expressions and individual gestures, (5) determination of detoxification type, and (6) provision of a postpurificatory dietary regimen after completion.

**Methods:**

The study will be conducted in 3 phases. The first is the preparation of standard operating procedure for TE data collection. The second is data annotation of detected vomiting events. All analyses will be conducted using Python libraries, including *scikit-learn* (version 1.3.2; developed by the scikit-learn contributors, Python Software Foundation), *TensorFlow* (version 2.14.0; Google Brain Team, Google LLC), and tools supported under Google Summer of Code 2023 (Google LLC), along with SPSS Statistics (version 26.0; IBM Corp) for statistical analysis. In the third phase, model performance will be evaluated using standard machine learning metrics, and agreement with expert assessments will be measured using the Fleiss κ statistic. This study is exploratory in nature. Thus, 50 volunteers will be targeted.

**Results:**

This is the first study of its kind, so to create the dataset, we prepared a standard operating procedure for TE event data collection. Data collection was completed in December 2025. Data annotation and preliminary model preparation are ongoing, with final testing and validation expected to be completed by December 2025. External testing in the health care setting is expected to be completed by February 2026.

**Conclusions:**

This work presents one of the first attempts to apply deep learning for objective analysis of the TE process in Ayurveda. By combining YOLOv9 for vomit detection and residual neural network for classification, the framework achieves promising accuracy in automated vomit identification. The results will demonstrate the potential of AI-assisted analysis in traditional medicine, although further clinical validation and expansion across multiple centers will be necessary before deployment in real-world settings.

## Introduction

### Background

The traditional Indian system of medicine promotes overall health and wellness through personalized therapies and a detoxifying process. Therapeutic emesis (TE), or *vamana karma*, is one of the bio-pentavalent purification procedures used to treat deranged *kapha* ailments that include metabolic, respiratory, and dermatological conditions such as psoriasis and eczema [1]. TE is conducted in 3 phases (ie, preparatory phase, main phase, and postpurificatory phase). In the main phase, volunteers are subjected to induced vomiting through the provision of emetic herbal medicines. After initiation of the process, the individual expels the vitiated content, concurrently evident through physical changes (concurrently evident include the onset of nausea, increased salivation, abdominal discomfort, sweating, piloerection, lacrimation, and the progressive expulsion of gastric contents, reflecting effective mobilization and elimination of morbid bio-humor) [2]. This procedure is carried out early in the morning and takes approximately 1 hour, although it sometimes takes more than an hour to complete.

Previously, TE has been assessed subjectively by trained physicians through visual inspection–based emesis indicators, such as patient gestures that include nausea, discomfort, and distress and vomitus characteristics such as color, consistency, volume, and odor. Nevertheless, dependence on human knowledge may introduce observer bias and interindividual variability, affecting reliability in clinical outputs [3]. Furthermore, extended therapy is often time-consuming and poses challenges for physicians in observing and documenting continuously, potentially creating inconsistency and reducing standardization. Studies suggest that human error is one of the most significant contributors to decreased productivity and quality. Multifactorial interaction among stress, repeated tasks, fatigue, and workplace circumstances impairs cognitive performance, attention, and decision-making accuracy [4]. Similarly, the variable rapid nature of emesis creates difficulties in manual tracking due to the rapid onset of the process, thus limiting real-time monitoring.

Research highlights that the integration of artificial intelligence (AI) techniques in image analysis for clinical decision support showcases their potential in diverse areas. Today’s health care practices have changed significantly with the help of AI and machine learning (ML), which help with more accurate diagnoses, custom interventions, and informed decisions [5,6]. Previous studies have substantiated that the use of AI-assisted imaging yields superior accuracy and specificity compared to traditional methods [7]. Further cutting-edge developments using YOLOv9, TensorFlow, and residual neural network (ResNet) models have yielded newer insights in scope for such assessment methods.

There have been many studies conducted on *vamana karma* focusing on its efficacy in diseases and biophysical parameters [8-10]. Presently, no study has been proposed to digitally analyze the procedure. This study has been designed to integrate for the first time an AI component for TE analysis. The primary objective is to support physicians in accurate interpretations of purification type and guiding patients to an appropriate postpurificatory diet. The primary aim is to assess appropriate signs and symptoms of TE through content analysis of vomitus by developing and validating an AI model using digital image processing and an AI algorithm. Additional objectives focus on developing a dataset of TE for training and validating an AI model, training the TE model for real-time assessment, and validating the performance of the developed AI model by comparing its assessments with those of expert Ayurvedic physicians.

### Research Imperative

In the traditional system of medicine, TE is a key intervention for body detoxification, which requires documentation of all evident changes during the process. Recent studies have underscored the adoption of the YOLOv9 approach for detecting abnormal crowd behavior to promote crowd safety [11]. Correspondingly, another study used the same methodology in traffic hazards for public safety using the GC-YOLOv9 algorithm [12]. Additionally, there are studies in which image processing was used for microscopic sputum analysis and the velocity distance support algorithm was used for sputum monitoring to detect phlegm stagnation [13,14]. Contemporarily, one study was conducted to automate emesis in *Suncus murinus* using a convolutional neural network and a self-attention mechanism [15]. Such studies draw attention to developing a model that can automate emetic episodes in traditional sciences. Moreover, the convergence of such approaches remains unexplored. Leveraging existing technologies could enable real-time monitoring for automation, enhance reliability, and improve patient outcomes.

## Methods

### Study Design

This is a prospective, observational, single-center study aimed at developing and validating an AI-based framework for the objective assessment of *vamana karma* (TE), a classical purification therapy in Ayurveda. The study adheres to the Standards for Reporting Diagnostic Accuracy–Artificial Intelligence reporting guidelines [16]. It will ensure data handling with model development, validation, and performance. A checklist has been provided in Multimedia Appendix 1.

### Study Setting

**Figure 1.**
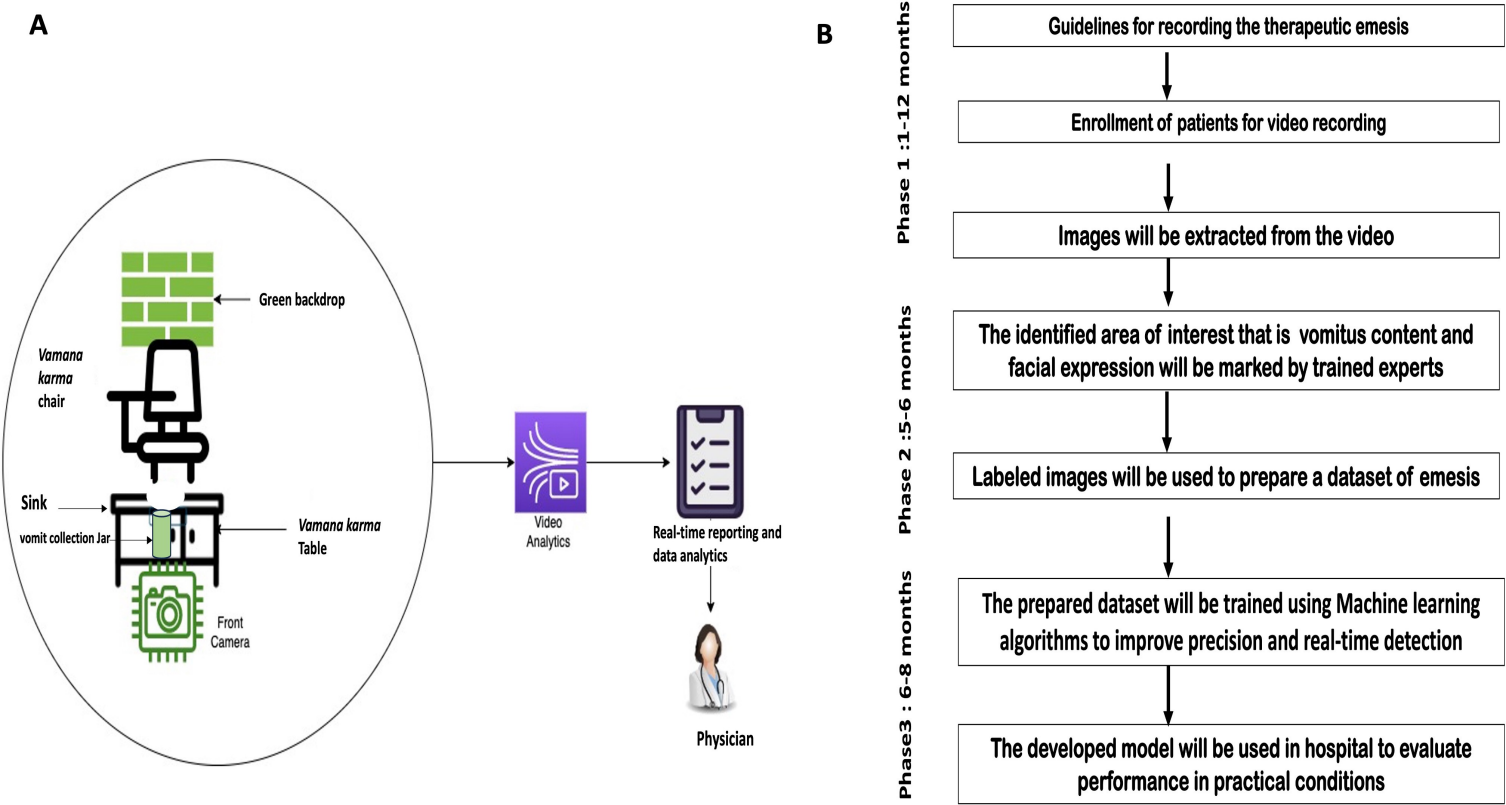
(A) Schematic representation of the data collection setup during *vamana karma* showing patient positioning, camera placement, vomit collection, and green backdrop for optimal video capture. (B) Study workflow and timeline illustrating the sequential phases of the research process, including data collection, manual annotation, machine learning model development, system optimization using trained datasets, and user testing for performance validation.

The study will be conducted at a tertiary Ayurveda research and teaching institute with an established *panchakarma* (bio-purification methods) center. A dedicated clinical setup will be designed to ensure uniform data collection across participants. The study workflow is depicted in Figure 1A and Figure 1B.

To achieve uniformity in data collection, we will adopt the following guidelines throughout the process:

- A monochromatic green backdrop will be used to optimize background segmentation for video analytics.
- Participants who undergo TE will be dressed in a green gown for the same purpose.
- Standardized artificial lighting will ensure uniform illumination and minimize the effect of varying light conditions.
- High-definition cameras (minimum 1080p resolution) will be positioned at forehead level facing the participant to record both the vomitus events and the patients’ facial expressions.
- Vomitus will be collected in transparent jars placed beneath the *vamana karma* chair setup.

### Participants

After providing informed consent, adult participants aged between 18 and 65 years will be enrolled in the study. Inclusion and exclusion criteria will be strictly adhered to for participant safety and data reliability. Inclusion criteria are adults clinically recommended for TE as per classic Ayurvedic guidelines for *kapha*-dominant conditions such as asthma, skin diseases, and metabolic disorders. Participants must be willing to consent to video recording during the procedure. Exclusion criteria include individuals with contraindications for *vamana karma*, including pregnancy, lactation, cardiac conditions, anemia, or known hypersensitivity to emetic medicine. Individuals unable or unwilling to provide informed consent will also be excluded.

### Sample Size

A total of 40 high-resolution videos (approximately 70 minutes each) were used, yielding over 10,000 frames annotated for vomit detection. This dataset size was considered adequate for training a deep learning model given the high temporal density of labeled events and the pretrained backbone used (YOLOv9 and ResNet). A preliminary learning curve analysis indicated performance saturation beyond this dataset size. Thus, for this study, we targeted 50 participants.

### Clinical Phases of TE

**Figure 2.**
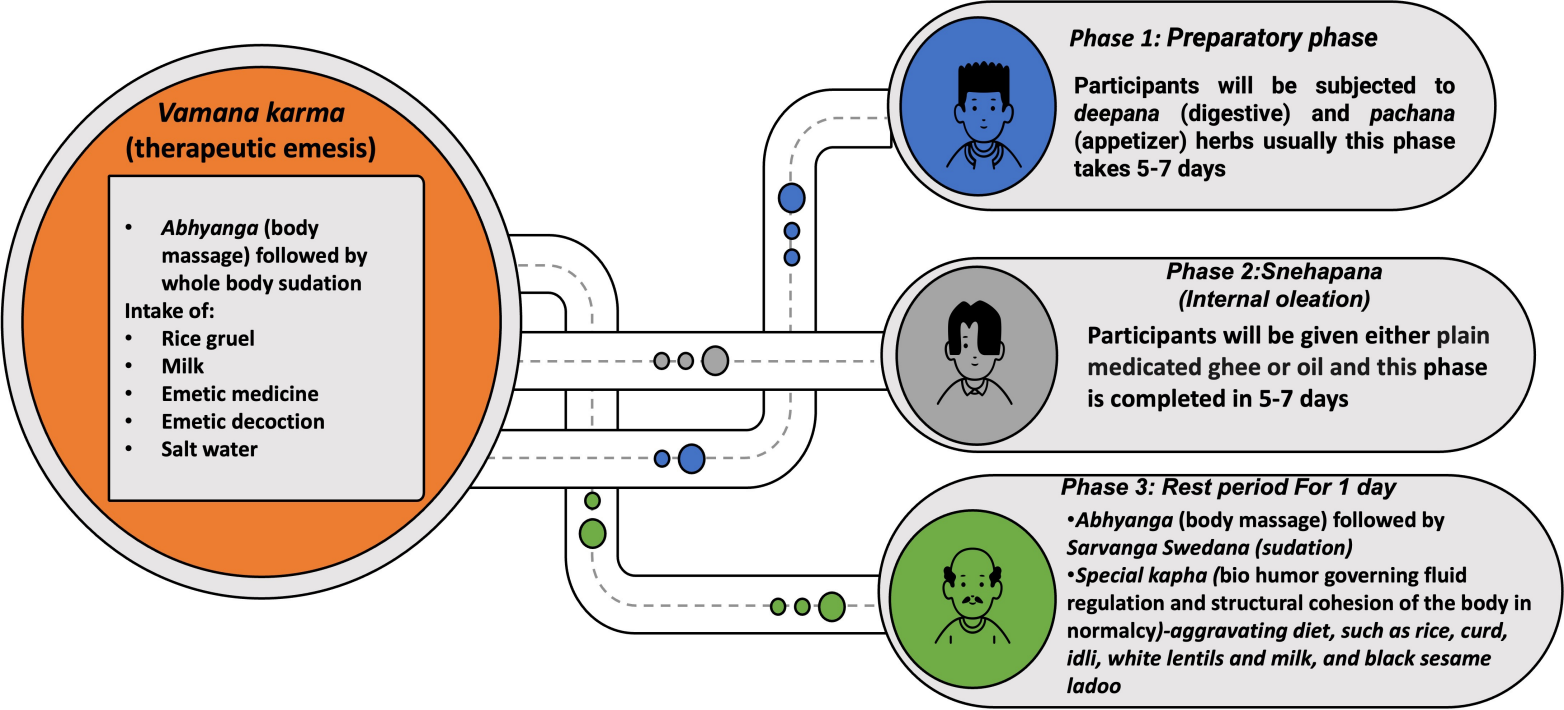
Procedural phases of *vamana karma*, including preparatory (*deepana*-*pachana*), internal oleation (*snehapana*), and rest period showing diet and therapeutic interventions.

The complete process will be conducted in 3 phases, as shown in Figure 2. Phase 1, also known as the preparatory phase (*purva karma*), starts with the administration of *deepana* (digestive stimulants) and *pachana* (digestive agents), followed by *snehapana* (internal oleation) for 5 to 7 days accompanied by *abhyanga* (massage) and *svedana* (sudation) for 1 day. In this 1-day period, a *kapha*-aggravating diet will be administered. Thereafter, the second or main phase (*pradhana karma*) starts on the *vamana karma* day with *abhyanga* (massage) and *svedana* (sudation). Participants will be allowed to take the following emetic formulations early in the morning: (1) rice gruel (consistency, color, and texture), (2) milk (2 L), (3) 8 g of medicine (madanphala powder; *Randia dumetorum* Linn.)+5 g of madhuyashti powder (*Glycyrrhiza glabra* Linn.)+2 g of vacha powder (*Acorus calamus* Linn.)+5 g of salt+honey (q.s.), (4) 30 g of madhuyashti kwath (decoction of *G glabra* Linn.) powder dissolved in 1 L of hot water, and (5) salt water (20 g of salt dissolved in 1 L of water; 1 glass=380 mL). The number, content, and force of vomitus bouts will be recorded. Vomit will be collected in calibrated jars for visual analysis and recorded via video.

In the third or postprocedure phase (*paschat karma*), after completion of *vamana karma*, depending on the type of purification, the participants will be gradually shifted to *samsarjana karma* (postdetoxification dietary regimen) to normalize the weak digestive fire.

Video data will be captured throughout the procedure to record vomitus events, facial expressions, and physical gestures. The data will form the primary source for model training and validation.

### Data Annotation

Video data will be processed through frame extraction and manual annotation to create labeled datasets for ML. More than 11,000 frames will be annotated for vomitus event detection, whereas approximately 700 to 800 cropped images will be classified as per classic parameters. All these annotations will serve as the ground truth for supervised learning and will be validated by experienced Ayurveda physicians to ensure accuracy. Data labeling details are listed in Table 1.

**Table 1.** Outcome parameters used for analysis and manual annotation. The outcome parameters constitute the core analytical content of the study and were derived from predefined clinical variables. All parameters were manually annotated and labeled by trained reviewers to ensure consistency and accuracy of the dataset used for analysis.

Outcome parameters	Labeled and description
Milk	Liquid, thin consistency and white color
Yavagu (rice milk, jaggery, and ghee)	Thick consistency; rice particles of brown color mixed with milk
Phanta	Water flow, thin consistency, and light yellow-brown color
Medicine	Thick pastelike consistency, less viscous, dark brown color, and less quantity with decreased flow
Salt water	Normal flow, watery consistency, and transparent
Pitta	Yellow color, thick consistency, normal flow, and much lower quantity
Facial expression analysis	The patients' facial gestures will be analyzed by mapping various features of the face, such as the eyebrows, eyes, and mouth, to the emotions of anger, fear, surprise, sadness, and happiness
Variations in the labeling of parameters	
Phanta+yavagu	Light yellowish-brown color and rice particles
Salt water+milk+medicine	Transparent; brown particles with a slight white color
Pitta+salt water	Water of a yellowish color
Pitta+medicine+rice	Yellow color, brown particle–like consistency, and rice particles
Pitta	Light yellow color
Rice particles	Thick consistency—particles the size of a small dot
Salt water+medicine	Water of a light brown color
Rice particles+medicine particles	Light brown color; medicine particles the size of minute dots
Milk mixed with medicine	Light brownish color
Milk+phanta	White color with light brown texture
Phanta+milk	Light yellowish-brown color; small milk particles
Phanta+pitta	Yellow color with a light yellowish-brown–colored liquid
Phanta+medicine	Light yellowish-brown color
Medicine+milk	White brown color; liquid, slightly thick consistency; and small particles with a white structure
Milk mixed with medicine	White appearance with a brown color texture
Milk+salt water	Transparent with white appearance
Phanta+medicine	Light yellowish-brown color, dark color, and more particles also present
Salt water+rice particles	Transparent water; rice particles appear dotted
Blood+milk	Red color mixed with white particles

### ML Pipeline

#### Overview

**Figure 3.**
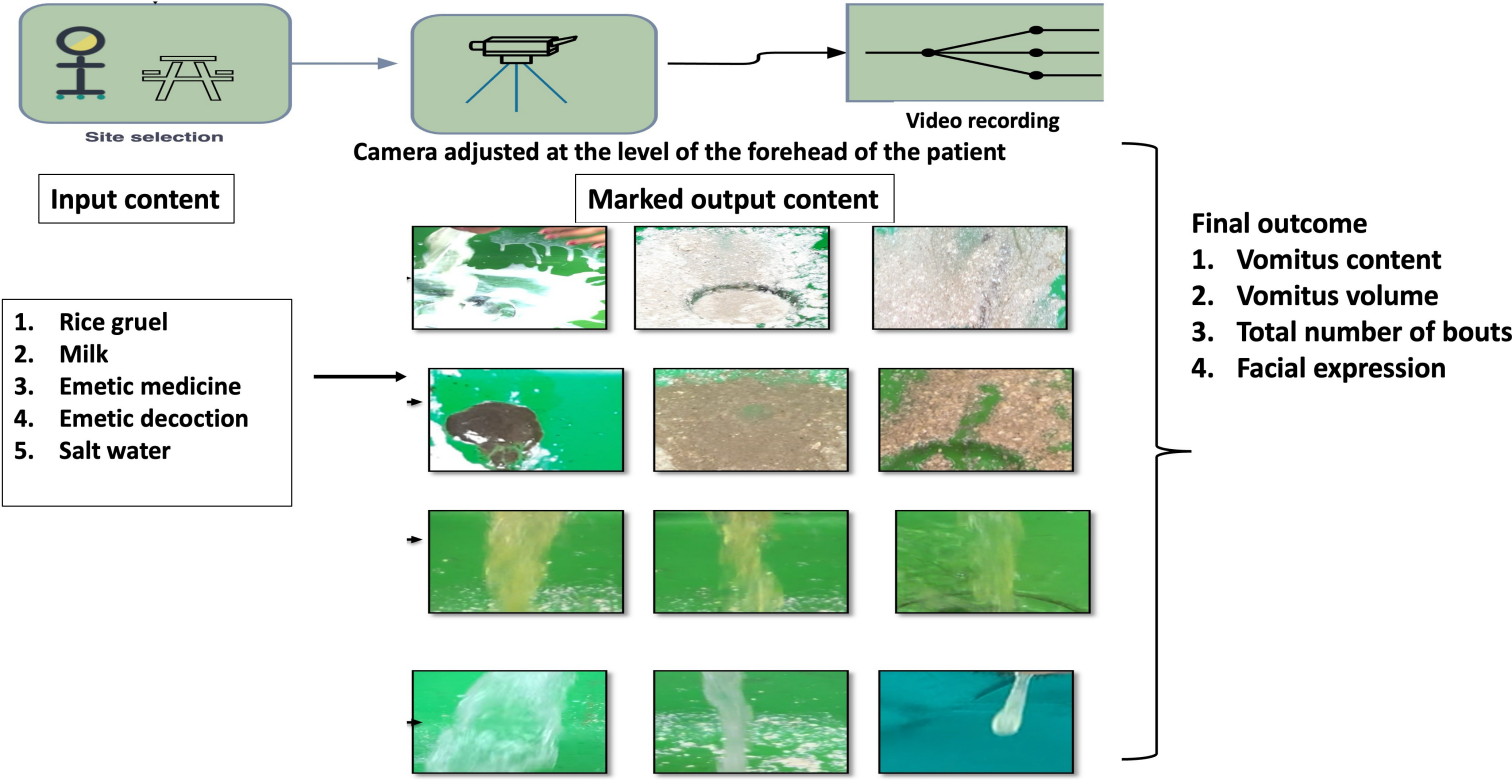
Flow diagram of input-output mapping for vomitus classification based on image analysis and machine learning data training pipeline.

The AI framework will integrate several ML components, as shown in Figure 3.

#### Object Detection

A YOLOv9 deep learning model will be trained to detect and localize vomitus streams in real-time video frames. The model will be trained on the annotated images and validated using k-fold cross-validation. Key performance metrics will include detection accuracy and precision-recall scores.

#### Content Classification

A convolutional neural network based on ResNet architecture will be developed for vomitus content classification. The model will categorize each vomitus image into one of the predefined classes. Data augmentation techniques will be applied to increase dataset variability and improve generalization.

#### Facial Expression and Gesture Analysis

Facial expression analysis will be conducted using DeepFace (Meta Platforms), an open-source facial recognition and analysis framework. Expressions such as disgust, sadness, tiredness, and anger will be detected. Physical gestures, including hand and eye movements, will also be analyzed using pose estimation models.

#### Temporal Analysis

Detected events, classified vomitus types, and facial expressions will be time-stamped and mapped across the procedure timeline. This will enable the correlation of emesis patterns and patient responses, facilitating a structured understanding of the *vamana karma* process.

### Statistical Analysis

Descriptive statistics will summarize participant demographics and baseline characteristics. ML model performance will be reported using accuracy, precision, recall, *F*_1_-score, and area under the receiver operating characteristic curve.

Model validation will use 5-fold cross-validation techniques to ensure robustness. Agreement between AI outputs and physician assessments will be evaluated using the Fleiss κ with standard interpretation thresholds of <0.40 for poor agreement, 0.40 to 0.75 for fair to good agreement, and 0.75 for excellent agreement.

All analyses will be conducted using Python libraries (scikit-learn [Google Summer of Code] and TensorFlow [Google Brain Team]) and the SPSS software (IBM Corp) for statistical analysis.

After training both the YOLOv9 and ResNet models, testing will be conducted in the following structured way. The dataset will be divided into 70% for training, 15% for validation, and 15% for testing. The test set will consist of unseen video samples containing vomiting and nonvomiting events to ensure unbiased evaluation. The YOLOv9 model will be tested on the test dataset to detect vomit regions frame by frame, output bounding boxes, and confidence scores. To test the complete pipeline, the full video will be passed through YOLOv9 followed by ResNet. The pipeline will be evaluated on frame-level accuracy (correct vomit identification across frames), event-level accuracy (correct identification of full vomiting events), and processing time per frame (frames per second) to measure real-time feasibility.

K-fold cross-validation (eg, *K*=5) will be performed to verify model robustness and reduce overfitting. Additionally, to prevent data leakage, participant-level splitting will be used during model evaluation. All frames from a given participant will be kept within a single fold to ensure that no individual’s data appear in both the training and test sets. This ensures realistic generalization performance.

To enhance clinical interpretability, gradient-weighted class activation mapping visualization will be explored on the ResNet classifier outputs to highlight the image regions contributing to vomit type classification. This step supports explainability and builds clinician trust in the AI’s decisions.

### Outcome Measures

The primary outcome is accuracy (or *F*_1_-score) of vomit event detection and classification at the participant level. Secondary outcomes are inference time per frame and classification consistency across participants.

### Ethical Considerations

Ethics approval for the study was obtained from the Institutional Ethics Committee of All India Institute of Ayurveda, New Delhi, India before initiation (325/19.12.2022/PhD-04/2022 [reissued in 2023]). All participants will provide written informed consent before recording after being informed about the study objectives, the voluntary nature of participation, and their right to withdraw at any time without consequences. Videos will be anonymized by removing identifiable facial features and stored in encrypted format (Advanced Encryption Standard–256) on a secure local server with access restricted to authorized researchers.

### Cultural Considerations

While the potential of AI integration in Ayurveda is significant, it also raises ethical and cultural challenges. AI-based evaluation of deeply traditional and personalized procedures may be viewed with skepticism by practitioners who value individualized clinical judgment. Hence, AI tools should be positioned as decision support systems rather than replacements for practitioner expertise. Transparent algorithms, patient consent, and data privacy must remain central to any implementation.

## Results

The study was initiated in January 2024 and is currently ongoing. Participant enrollment and video data collection began in February 2024, with a target of enrolling 50 patients by March 2025. Procedures for annotating data and training ML models are being carried out. Data analysis is scheduled to commence in May 2025, and the final results are expected to be available by December 2025.

## Discussion

### Expected Findings

The study seeks to test a novel way to assess TE by using an AI model. The proposed framework will evaluate the outcomes more accurately and ease for the physician by automating the vomiting event. Once validated, this framework could serve as a model for the application of AI in other traditional medicine procedures, thereby contributing to the broader field of integrative and digital health. Previous studies in medical imaging and diagnostic AI have established the capacity of ML algorithms to identify subtle patterns in biomedical data—such as radiographs, endoscopic images, and pathology slides—that often escape human perception. In traditional systems of medicine, similar AI-based models have been used for pulse diagnosis, tongue image interpretation, and facial feature analysis, suggesting the need for deployment [17-21]. However, no prior work has attempted this in the domain of vomitus analysis, leaving a critical research gap that needs to be addressed. The framework can be generalized to other *panchakarma* procedures or Ayurvedic practices involving visual inspection provided that adequate domain-specific annotations are available. Its modular design allows for retraining with site-specific data for broader clinical adoption. The study establishes the foundation for image-based pattern recognition models that could one day assist practitioners in evaluating the *shuddhi lakshana* (signs of purification) objectively.

### Strengths of the Study

The study will help enhance diagnostic precision and documentation. Moreover, it enables longitudinal data collection that may reveal new correlations among patient profiles, therapy outcomes, and procedural parameters, which were otherwise difficult to quantify manually.

### Limitations

The study’s shortcomings include a small sample size, possible observer bias in vomitus labeling, and limited generalizability due to single-center data. Data will be collected in a controlled clinical setting, limiting real-world diversity. TE is a seasonal therapy traditionally administered during specific periods of the year, which naturally limits the availability of eligible participants and, consequently, the sample size for ML model development and validation. Furthermore, the digital interpretability of Ayurvedic features such as *pitta* (bio humor responsible for metabolic activities) and *aushadhi* (medicine) remains challenging. Despite these limitations, the study is an important step toward developing a validated framework for combining AI with traditional assessments. More comprehensive multicentric research is necessary to validate the proposed concept across diverse populations and practice settings.

### Future Directions

Future work will focus on validating the framework across larger, more diverse patient populations and exploring its application in real-time clinical decision-making. Although this work focuses on model development and internal validation, future studies will involve clinical validation across multiple *panchakarma* centers to assess model robustness under real-world variations in lighting, camera positioning, and practitioner styles.

### Conclusions

This work presents one of the first attempts to apply deep learning for objective analysis of the *vamanakarma* process in Ayurveda. By combining YOLOv9 for vomit detection and ResNet for classification, the framework achieves promising accuracy in automated vomit identification. The results will demonstrate the potential of AI-assisted analysis in traditional medicine, although further clinical validation and expansion across multiple centers will be necessary before deployment in real-world settings.

## Supplementary material

10.2196/79875 Multimedia Appendix 1 Supplementary table S1.

## Abbreviations

AI: artificial intelligence
ML: machine learning
ResNet: residual neural network
TE: therapeutic emesis
