# Supplementary material for: Using AI Algorithms and Machine Learning in the Analysis of a Bio-Purification Method (Therapeutic Emesis, Known as “Vamana Karma”): Protocol for a Mixed Methods Study
Source: JMIR Res Protoc. 2026 Feb 3;15:e79875. doi: 10.2196/79875 (PMC12867470; doi:10.2196/79875)
Supplement: Multimedia Appendix 1 [file resprot-v15-e79875-s001.docx]

Table S1. STARD AI Checklist.

| **S.No.** | **Section** | **Description** |
| --- | --- | --- |
| **1** | **Identification as an AI Diagnostic Study** | The study will follow the STARD-AI framework for transparent reporting for prediction accuracy involving artificial intelligence |
| **2** | **Scientific and Clinical Background** | Therapeutic emesis (T.E), known as vamana karma, is a classical method of detoxification performed to eliminate vitiated kapha ailments primarily from the body. This complete process assessment depends on physicians’ visual assessments of vomitus features and patient responses, introducing subjectivity and interobserver variability. Moreover, this method requires more than continuous monitoring; thus, a physician can sometimes lead to human errors, resulting in missed expelled content or complications. This AI model is proposed to monitor T.E to observe visual changes, i.e. patient vomitus content and gestures, to provide better. clinical outcomes. This approach has been explored for the first time in the traditional system of medicine. |
| **3** | **Study Objectives and Hypothesis** | **To assess appropriate signs and symptoms of therapeutic emesis through content analysis of vomitus by developing and validating an AI model using digital image processing and an AI algorithm.** |
| **4** | **Study Population** | **Patient undergo for Therapeutic emesis. The nature of study is exploratory thus 50 patients video will be recorded.** |
| **5** | **Index Test (AI Algorithm)** | A convolutional neural network (CNN)-based architecture (ResNet/TensorFlow/YOLO v9) was used for vomitus image classification and feature extraction. |
| **6** | **Reference Standard** | Visual evaluation of vomitus characteristics (colour, consistency, volume, odor) by experienced Ayurvedic physicians will served as the reference standard. |
| **7** | **Blinding** | **Not applicable** |
| **8** | **Data preprocessing and annotation** | Images were cleaned, annotated, and validated using a consensus approach. Annotation consistency was ensured through inter-rater reliability analysis |
| **9** | **Statistical Analysis** | Descriptive statistics will summarise participant demographics and baseline characteristics. Machine learning model performance will be reported using accuracy, precision, recall, F1-score, and area under the receiver operating characteristic (ROC) curve.  Model validation will employ 5-fold cross-validation techniques to ensure robustness. Agreement between AI outputs and physician assessments will be evaluated using Fleiss' Kappa, with standard interpretation thresholds:   - <0.40: Poor agreement - 0.40–0.75: Fair to good agreement - 0.75: Excellent agreement   All analyses will be conducted using Python libraries (scikit-learn, TensorFlow) and SPSS software for statistical analysis.  a) Testing the Dataset and Model- After training both YOLOv9 and ResNet models, testing will be done in the following structured way:  1. Dataset Split- The dataset will be divided into: 70% for training, 15% for validation and 15% for testing. The test set will consist of unseen video samples containing vomiting and non-vomiting events to ensure unbiased evaluation.  b) YOLOv9 Model Testing (Detection Stage)- The YOLOv9 model will be tested on the test dataset to: Detect vomit regions frame-by-frame, Output bounding boxes and confidence scores.  Metrics Used: mAP (Mean Average Precision) for detection accuracy. Precision, Recall, and F1-score to evaluate true positives and false detections. IoU (Intersection over Union) threshold of 0.5 will be used to determine correct detections.  c) ResNet Model Testing (Classification Stage)- Each detected vomit region (cropped from the YOLOv9 output) will be passed to the ResNet classifier. The ResNet model will classify the region into predefined categories (e.g., vomit type, severity level, etc.).  Metrics Used: Accuracy, Precision, Recall, F1-score, and Confusion Matrix for performance evaluation , ROC-AUC curve (if there are multiple classes).  d) End-to-End Pipeline Testing -To test the complete pipeline, the full video is passed through YOLOv9 followed by ResNet. The pipeline will be evaluated on:  Frame-level accuracy: Correct vomit identification across frames.  Event-level accuracy: Correct identification of full vomiting events.  Processing time per frame (FPS) to measure real-time feasibility |
| **10** | **Model Validation** | Cross-validation - K-Fold Cross-Validation (e.g., K=5) will be performed to verify model robustness and reduce overfitting. |
| **11** | **Results Summary** | The study was initiated in January 2024 and is currently ongoing. Participant enrolment and video data collection began in February 2024, with a target of enrolling 50 patients by March 2025. Procedures for annotating data and training machine learning models are being carried out. Data analysis is scheduled to commence in May 2025, and the final results are expected to be available by December 2025. |
| **12** | **Limitations** | The study's shortcomings include a small sample size, possible observer bias in vomitus labelling, and limited generalizability due to single-centre data.. Data will be collected in a controlled clinical setting, limiting real-world diversity. T.E is a seasonal therapy traditionally administered during specific periods of the year, which naturally limits the availability of eligible participants and, consequently, the sample size for machine learning model development and validation. Furthermore, digital interpretability of Ayurvedic features like pitta, aushadhi etc., remains challenging. Despite these limitations, the study is an important step toward developing a validated framework for combining AI with traditional assessments. More comprehensive multicentric research is necessary to validate the proposed concept across diverse populations and practice settings. |
| **13** | **Implications** | This study introduces a conceptual framework for integrating AI and ML algorithms into the clinical evaluation of Vamana, marking the first step toward digitising traditional bio-purification procedures in Ayurveda. The proposed approach demonstrates that objective data extraction and computational analysis are feasible without compromising traditional diagnostic principles. Such integration can advance evidence-based Ayurveda by supporting objective evaluation, improving reproducibility, and enhancing the credibility of traditional therapies within modern healthcare systems. |
